# Comparison of genotyping methods and toxin gene profiles of *Staphylococcus aureus* isolates from clinical specimens

**DOI:** 10.1590/1678-4685-GMB-2022-0321

**Published:** 2023-12-22

**Authors:** Mariana Andrade-Figueiredo, Ana Carolina de Oliveira Luz, Vladimir da Mota Silveira, Tereza Cristina Leal-Balbino

**Affiliations:** 1 Centro Universitário Boa Viagem Wyden (UNIFBV Wyden), Departamento de Biomedicina, Recife, PE, Brazil.; 2 Fundação Oswaldo Cruz, Instituto Aggeu Magalhães (IAM), Departamento de Microbiologia, Recife, PE, Brazil.; 3 Universidade de Pernambuco (UPE), Departamento de Biologia, Garanhuns, PE, Brazil.

**Keywords:** Staphylococcus aureus, MRSA, MSSA, genotyping, exotoxins

## Abstract

*Staphylococcus aureus* is a frequent cause of infections worldwide. Methicillin-resistant *S. aureus* (MRSA) is one of the main causes of Gram-positive infections, and methicillin-susceptible strains (MSSA) primarily colonize and infect community hosts. Multiple virulence factors are involved, with toxins playing a significant role in several diseases. In this study, we assess the prevalence of toxin genes in 89 *S. aureus* clinical isolates (31 MRSA and 58 MSSA). We evaluated the discriminatory power of the association of internal transcribed spacer-PCR (ITS-PCR) and 3’- end *coa* gene ( *coa*-PCR) when compared with other more commonly used and costly techniques. The isolates showed a high level of genetic diversity, and toxins were found in all the isolates. While most toxin classes displayed no statistically significant correlations and were equally distributed in isolates regardless of their resistance status, classic enterotoxins ( *sea-see*) showed a positive correlation with MSSA isolates. The combination of *coa*-PCR with ITS-PCR showed a discriminatory index of 0.84, discriminating 22 genotypes that agree with previously determined data by PFGE and MLST. This association between the two PCR-based methods suggests that they can be useful for an initial molecular epidemiological investigation of *S. aureus* in hospitals, providing significant information while requiring fewer resources.

## Introduction

Methicillin-resistant *Staphylococcus aureus* (MRSA) and methicillin-sensitive *S. aureus* (MSSA) are major pathogens associated with serious infections in both hospitals and communities worldwide ( Tong et al., 2015; Arias et al., 2018; Junie et al., 2018; Lakhundi and Zhang, 2018). In Latin American countries like Brazil, Bolivia, Chile, and others, more than 50% of *S. aureus* isolates are already categorized as MRSA and can be considered resistant to most β-lactams ( Lee et al., 2018).

Exotoxins produced by *S. aureus* can cause diarrhea that is either related or not to the use of antibiotics, gastroenteritis, or food poisoning ( Ortega et al., 2010; Pinchuk et al., 2010). This pathogen is also connected to severe illnesses like Toxic Shock Syndrome (TSS) and Scalded Skin Syndrome (SSS), which are regarded as superantigens and caused by toxin production ( Ladhani et al., 1999; Dinges et al., 2000). The group of superantigens includes staphylococcal enterotoxins (SE), toxic shock syndrome toxin (TSST), and exfoliative toxins (ET) ( Portillo et al., 2013).

For epidemiological purposes, it is crucial to identify the distribution of clinical isolates, and genotyping has emerged as a key tool in medical investigations to identify strain origin, clonal relatedness, and outbreak epidemiology ( Olive and Bean, 1999; Arias et al., 2018). Genotyping methods often involve applying different molecular techniques based on PCR, sequencing, or genomic macrorestriction ( Rodríguez-Noriega et al., 2010). 

By combining these methods, strains can be classified into different lineages, or clones. Some clones of *S. aureus* are known as epidemic clones, meaning they are descendants of the same ancestor and are widely distributed among different countries. Some of these lineages, such as the Brazilian Epidemic clone (BEC), the Pediatric clone (PC), and the Cordobes/Chilean clone, have already been identified as native to Latin America and are useful for describing the various genetic backgrounds of *S. aureus* ( Zurita et al., 2016). 

Since few data have been published in Brazil, particularly in the Northeast region, our goal was to investigate the exotoxin gene profile for MRSA and MSSA isolates from patients admitted to hospitals in Recife city, Pernambuco state, Brazil. Additionally, we aimed to evaluate the ability of coagulase gene typing ( *coa*-PCR) and ribosomal 16S-23S internal transcribed spacer (ITS-PCR) to distinguish isolates and clones from clinical onsets in comparison to techniques more frequently used for molecular epidemiology studies of *S. aureus*, such as pulsed-field gel electrophoresis (PFGE), multilocus sequence typing (MLST), *spa* typing, and Staphylococcal Cassette Chromosome *mec* (SCC *mec*) genotyping.

## Material and Methods

### Ethics statement

The project was approved by the Oswaldo Cruz Foundation Health Research Ethics Committee, Aggeu Magalhães Institute, IAM/Fiocruz, Brazil (CEP: 0024.0.095.000-07). 

### Bacterial isolates and DNA extraction

In this investigation, 89 *S. aureus* isolates were obtained on spontaneous demand from hospitals in Recife that treat patients from different parts of Pernambuco ( Table S1). Prior to the present study, these isolates were typed using PFGE, MLST, *spa*, phenotypic identification of resistance to cefoxitin, and SCC *mec* genotyping ( Andrade-Figueiredo and Leal-Balbino, 2016). 

Genomic DNA was extracted using the automated NucliSens-easyMAG (bioMérieux, Durham, NC), and PCR for toxigenic genes and molecular typing methods, designed for the present work, were performed on a GeneAmp PCR System 9700 (Applied Biosystems, Foster City, CA). 

### Detection of staphylococcal toxins genes

Using the primers given in Table 1, we examined the toxic shock syndrome toxin gene ( *tst*), two exfoliative genes ( *eta* and *etb*), and 14 staphylococcal enterotoxin (se) genes.

**Table 1 -:** Oligonucleotides used for the detection of exotoxin genes.

Toxin	Gene	Primer	Sequence (5’→ 3’)	Fragment size (pb)	Reference
SEA	*sea*	SEA-3b	CCTTTGGAAACGGTTAAAACG	127	Becker et al., 1998
		SEA-4b	TCTGAACCTTCCCATCAAAAAC		
SEB	*seb*	SEB-1c	TCGCATCAAACTGACAAACG	477	Becker et al., 1998
		SEB-4b	GCAGGTACTCTATAAGTGCCTGC		
SEC	*sec*	SEC-3b	CTCAAGAACTAGACATAAAAGCTAGG	271	Becker et al., 1998
		SEC-4b	TCAAAATCGGATTAACATTATCC		
SED	*sed*	SED-3b	CTAGTTTGGTAATATCTCCTTTAAACG	319	Becker et al., 1998
		SED-4b	TTAATGCTATATCTTATAGGGTAAACATC		
SEE	*see*	SEE-3b	CAGTACCTATAGATAAAGTTAAAACAAGC	178	Becker et al., 1998
		SEE-2c	TAACTTACCGTGGACCCTTC		
TSST-1	*tst*	TST-3	AAGCCCTTTGTTGCTTGCG	445	Becker et al., 1998
		TST-6	ATCGAACTTTGGCCCATACTTT		
ETA	*eta*	ETA-3b	CTAGTGCATTTGTTATTCAAGACG	119	Becker et al., 1998
		ETA-4b	TGCATTGACACCATAGTACTTATTC		
ETB	*etb*	ETB-3b	ACGGCTATATACATTCAATTCAATG	262	Becker et al., 1998
		ETB-4b	AAAGTTATTCATTTAATGCACTGTCTC		
SEG	*seg*	SEG-1	ACGTCTCCACCTGTTGAAGG	400	Rosec and Gigaud, 2002
		SEG-2	TGAGCCAGTGTCTTGCTTTG		
SEH	*seh*	SEH-1	TCACATCATATGCGAAAGCAG	357	Rosec and Gigaud, 2002
		SEH-2	TAGCACCAATCACCCTTTCC		
SEI	*sei*	SEI-1	GGTGATATTGGTGTAGGTAAC	454	Omoe et al., 2002
		SEI-2	ATCCATATTCTTTGCCTTTACCAG		
SEJ	*sej*	SEJ-1	CAGCGATAGCAAAAATGAAACA	426	Rosec and Gigaud, 2002
		SEJ-2	TCTAGCGGAACAACAGTTCTGA		
SEK	*sek*	SEK-1	CACAGCTACTAACGAATATC	378	Chiang et al., 2006
		SEK-2	TGGAATTTCTCAGACTCTAC		
SEL	*sel*	SEL-1	CATACAGTCTTACTAACGG	275	Chiang et al., 2006
		SEL-2	TTTTCTGCTTTAGTAACACC		
SEM	*sem*	SEM-1	CTTGTCCTGTTCCAGTATC	329	Chiang et al., 2006
		SEM-2	ATACGGTGGAGTTACATTAG		
SEN	*sen*	SEN-1	ATTGTTCTACATAGCTGCAA	682	Blaiotta et al., 2004
		SEN-2	TTGAAAAAACTCTGCTCCCA		
SEO	*seo*	SEO-1	AGTCAAGTGTAGACCCTATT	534	Blaiotta et al., 2004
		SEO-2	TATGCTCCGAATGAGAATGA		

Two multiplex PCR were performed, one for *sea-see* genes and another for *tst*, *eta* and *etb* genes. Uniplex PCRs were designated for *seg* to *seo* genes. PCR uniplex assays were prepared to a final volume of 50 µL, containing 40 ng chromosomal DNA, 0.8 mM of deoxynucleotide triphosphates, 1X PCR buffer, 1.5 mM MgCl_2_, 1 U *Taq* DNA polymerase (Promega, Madison, WI, USA) and 20 µM of each oligonucleotide. Amplification happened as follows: 95 ºC for 2 min, then 30 cycles of 95 ºC for 1 min, 55 ºC for 1 min and 72 ºC for 1 min. Products were separated by electrophoresis through 1.5% agarose gel. 

Multiplex assays were prepared to a final volume of 50 µL, containing 40 ng chromosomal DNA, 1 mM of deoxynucleotide triphosphates, 1X PCR buffer, 3 mM MgCl_2_, 1.5 U *Taq* DNA polymerase (Promega, Madison, WI, USA) and 20 µM of each oligonucleotide. Amplification consisted of denaturation 95 ºC for 2 min, then 30 cycles of 95 ºC for 1 min, 60 ºC for 1 min and 72 ºC for 2 min. Products were separated by electrophoresis through 1.5% agarose gel. 

The following *S. aureus* strains were used as positive controls: FRI722 and FRIS6 (for *sea* and *seb* genes, respectively), FRI361 ( *sec*, *sed*, *seg*, *sei* and *sej* genes), and FRIMN8 ( *tst* gene), provided by Food Research Institute (Madison, Wiscosin, EUA), 1SB and 3SB ( *sek, sel* and *sem*), MRSA41 ( *sen* and *seo*) and CR6 ( *seh*) (Aggeu Magalhães Institute, laboratory collection, Recife, PE, Brazil).

Toxin profiling was done by converting the toxin gene data into a binary matrix. For each isolate, a concatenation of these data yielded a binary profile resembling a barcode sequence. Table S2 has a detailed listing of these data.

### *coa*-PCR

The coagulase gene typing was performed as previously described ( Aarestrup et al., 1995). PCR assays were prepared to a final volume of 50 µL, containing 40 ng of chromosomal DNA, 0.8 mM of deoxynucleotide triphosphates, 1X PCR buffer, 2.5 mM MgCl_2_, 1 U *Taq* DNA polymerase (Promega, Madison, WI, USA), and 20 µM of each oligonucleotide. Amplification was made up of an initial denaturation of 95 ºC for 2 min, then 30 cycles of 95 ºC for 1 min, annealing at 55ºC for 1 min and extension at 72 ºC for 1 min. Products were submitted to electrophoresis through 1.5% agarose gel. *S. aureus* strain ATCC 25923 was used as a positive control.

### ITS-PCR

Amplification of the 16S-23S intergenic spacer region (ITS-PCR) employed a single pair of primers, as previously described ( Jensen et al., 1993). PCR assays were prepared to a final volume of 50 µL, containing 40 ng chromosomal DNA, 1 mM of deoxynucleotide triphosphates, 1X PCR buffer, 3 mM MgCl_2_, 1.5 U *Taq* DNA polymerase (Promega, Madison, WI, USA) and 20 µM of each oligonucleotide (Jensen *et al.*, 1993). Amplification was made up of 95 ºC for 5 min, then 30 cycles of 95 ºC for 1 min, 55 ºC for 1 min and 72 ºC for 2 min. Electrophoresis was performed using a 2% agarose gel. For quality assurance, *S. aureus* strains ATCC 33591 and ATCC 25923 were employed.

### Sequencing

A random sample of purified PCR products from toxigenic genes, ITS-PCR and *coa*-PCR (Purelink PCR purification kit, Invitrogen, Carlsbad, CA, USA) were chosen for DNA sequencing using the Big Dye Terminator Kit v3.1 and an ABI 3730xl DNA analyzer (Applied Biosystems, Foster City, CA, USA) in order to confirm only the specificity of the amplified genes. The PureLink PCR purification kit was provided by Invitrogen, Carlsbad, California, USA (Applied Biosystems, Foster City, CA, USA). The nucleotide sequences obtained were compared with the *S. aureus* sequence database in the GenBank through BLAST (http://www.ncbi.nlm.nil.gov).

### Discriminatory analysis

In order to compare the discriminatory index of typing methods, we used the formula described by Hunter and Gaston (1988): *DI = 1 - [1 / N(N - 1)] Σs nj(nj - 1)*, where *N* is the total number of isolates in this population, *s* is the total number of different types, and *nj* is the number of isolates representing each type. This formula is based on the probability that two unrelated strains taken from the population sample will be placed in different types of groups.

### Statistical analysis

We investigated any connections between the presence of toxins and the genotypes identified by Coagulase/ITS-PCR, as well as between the presence of toxins and the resistance status (either MRSA or MSSA). We used the Jamovi software’s Pearson’s correlation test, and only results with a p value of 0.05 or lower were considered statistically significant ( Jamovi, 2023).

## Results

### Toxigenic profiles of *Staphylococcus aureus* isolates

None of the 89 isolates carried *sed*, *see,* or *etb*, but all isolates were positive for at least three of the investigated genes. The most frequent toxin gene was *seg,* presented in 88/89 (99%) isolates, followed by *sem* 81/89 (91%), *seo* 80/89 (90%), *sen* 78/89 (88%) and *sei* 74/89 (83%), all members of the enterotoxin gene cluster ( *egc*). Six isolates (Sa08, Sa32, Sa80, Sa82, Sa87, and Sa88) were positive for the *tst* gene, and only one (Sa17) harbored *eta*. In one isolate (Sa87) from an ICU patient in Hospital 2, 12 of the 17 genes under investigation were found.

Statistically, however, the only relevant correlation happened between the MSSA group and classical enterotoxins. 19 MSSA isolates carried a sum of 21 genes, while MRSA isolates carried only one ( *sea*, in isolate Sa86 - toxin profile 33) ( Table 2).

**Table 2 -:** Pearson’s correlation between toxin gene counts and isolates’ resistance status (MRSA or MSSA).

		Resistance status
Toxin total count	Pearson’s R	-0.182
	p-value	0.088
ET^a^ count	Pearson’s R	-0.078
	p-value	0.468
TSST^b^ count	Pearson’s R	0.103
	p-value	0.339
Classic se^c^ count	Pearson’s R	-0.327
	p-value	0.002 ^*^
*egc* ^*d*^ count	Pearson’s R	0.035
	p-value	0.743
se^c^ count	Pearson’s R	-0.125
	p-value	0.242

^*^ statistically significant p-value

^a^ET = Exfoliative toxins;

^b^TSST = Toxic shock syndrome toxin;

^c^se = Staphylococcal enterotoxin;

^d^egc = Enterotoxin gene cluster.

All strains, regardless of susceptibility, carried at least two *egc*-related genes. A complete set of the *egc*, consisting of *seg, sei, sem, sen*, and *seo*, was found in 63/89 (71%), of which 22 are MRSA and 41 MSSA.

Isolates were subdivided into 45 genetic profiles based on their toxigenic content, and these genotypes were called toxin profiles ( Table S2). The 31 MRSA isolates exhibited 16 profiles, while the 58 MSSA isolates were associated with 39, with both groups sharing 10 of these toxin profiles. The toxin profile 22 - representing a complete *egc* in addition to genes *sej*, *sek,* and *sel* - was the most prevalent genotype, occurring in 10 isolates (10/89 - 16%; 5 MRSA and 5 MSSA). For both groups, toxin profile 22 was also the most frequent, followed by 17, which had four representatives in each group and corresponds to a complete *egc* in addition to gene *sek*.

### PCR-based typing methods

**Figure 1 -:**
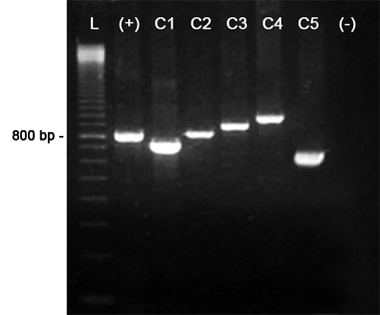
Agarose gel electrophoresis of Coagulase-PCR obtained with *Staphylococcus aureus* strains. L = 100 bp DNA ladder; (+) = positive control ~800 bp ( *S. aureus* ATCC 25923); C1 = coagulotype 1 ~730 bp (strain Sa22); C2 = coagulotype 2 ~810 bp (strain Sa41); C3 = coagulotype 3 ~890 bp (strain Sa45); C4 = coagulotype 4 ~970 bp (strain Sa44); C5 = coagulotype 5 ~650 bp (strain Sa88); (-) = negative control.

**Figure 2 -:**
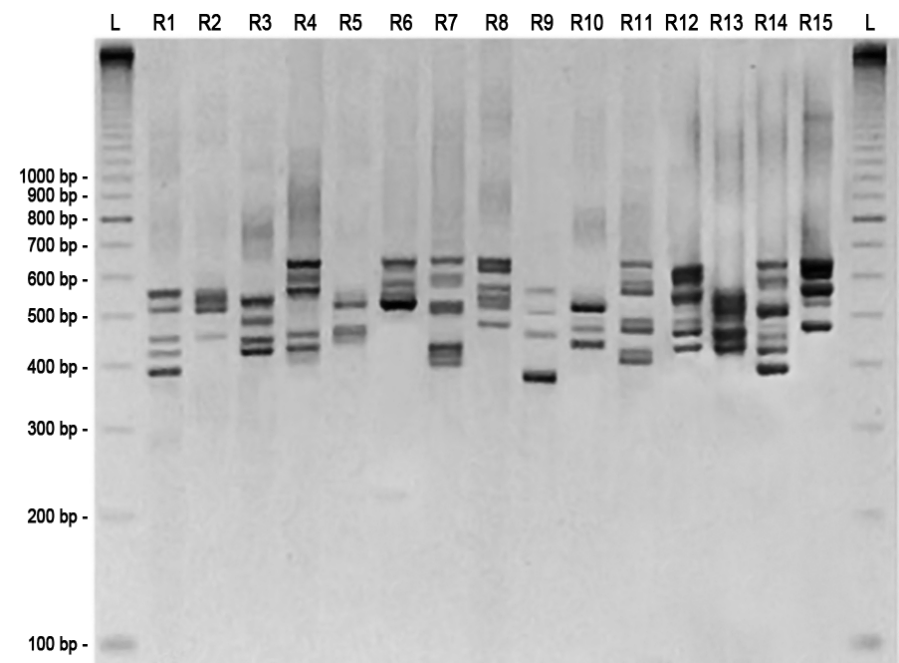
Agarose gel electrophoresis of ITS-PCR obtained with *Staphylococcus aureus* isolates. L = 100 bp DNA ladder; R1 = ribotype 1 (strain Sa81); R2 = ribotype 2 (strain Sa89); R3 = ribotype 3 (strain Sa04); R4 = ribotype 4 (strain Sa45); R5 = ribotype 5 (strain Sa08); R6 = ribotype 6 (strain Sa09); R7 = ribotype 7 (strain Sa26); R8 = ribotype 8 (strain Sa24); R9 = ribotype 9 (strain Sa28); R10 = ribotype 10 (strain Sa32); R11 = ribotype 11 (strain Sa61); R12 = ribotype 12 (strain Sa83); R13 = ribotype 13 (strain Sa84); R14 = ribotype 14 (strain Sa85); R15 = ribotype 15 (strain Sa88).

Coagulase genotyping analysis revealed five coagulotypes, according to the amplified segment, denominated as C1= ~730 bp (corresponding to 4 repeats), C2= ~810 bp (corresponding to 5 repeats), C3= ~890 bp (corresponding to 6 repeats), C4= ~970 bp (corresponding to 7 repeats) and C5 = ~650 bp (corresponding to 3 repeats). *S. aureus* ATCC 25923 amplified a fragment of ~810 bp ( Figure 1). In ITS-PCR reactions were observed 3 to 8 fragments with approximate sizes of 380 to 650 bp. Based on the amplification patterns, isolates were classified into 15 types, designated in this study as R1-R15 ( Figure 2).

The association between *coa*-PCR and ITS-PCR (C/R) analysis revealed 22 genotypes ( Table 3). The discriminatory index obtained by this combination was 0.84, similar to the index obtained by MLST (0.86) and *spa*-typing (0.89) but lower than the index obtained by PFGE (0.99). The 22 genotypes and the toxins under investigation did not show any statistically significant correlations.

**Table 3:** Molecular and toxigenic profiles of 89 *Staphylococcus aureus* isolates from hospitals in Recife, Brazil.

C/R genotype	coa- PCR	ITS- PCR	Strain	OXA/ CFO^a^	SCC mec	MLST	PFGE type	PFGE cluster	spa type	E/P Clone^b^	**ET** ^c^		TSST^d^	**Classic se** ^e^					**egc** ^*f*^					**se**				**Toxin profile**
											*eta*	*etb*	*tst*	*sea*	*seb*	*sec*	*sed*	*see*	*seg*	*sei*	*sem*	*sen*	*seo*	*seh*	*sej*	*sek*	*sel*	
1	C5	R15	Sa88	S	-	ST15	55	-	t084	-	-	-	+	-	-	-	-	-	+	+	+	+	+	+	+	+	+	41
2	C1	R2	Sa22	**R**	III	ST239	3	A	t037	BEC	-	-	-	-	-	-	-	-	+	-	+	+	+	-	-	+	+	8
3	C1	R3	Sa68	S	-	ST25	26	-	t1102	-	-	-	-	-	+	-	-	-	+	+	+	+	+	-	+	+	-	30
	C1	R3	Sa65	S	-	ST30	65	H	t433	USA1100^j^	-	-	-	-	-	-	-	-	+	+	+	+	+	+	-	+	+	24
	C1	R3	Sa73	**R**	III	ST239	11	B	t037	BEC	-	-	-	-	-	-	-	-	+	-	-	-	+	-	+	-	+	2
	C1	R3	Sa77	S	-	ST333	51	G	t279	-	-	-	-	-	-	-	-	-	+	+	+	-	-	-	+	-	+	11
	C1	R3	Sa74	S	-	ST718	68	-	t1442	-	-	-	-	-	-	-	-	-	+	-	-	-	+	-	+	-	+	2
	C1	R3	Sa75	S	-	ST718	69	-	t1442	-	-	-	-	-	-	-	-	-	+	-	-	-	+	-	+	+	+	3
4	C1	R4	Sa39	**R**	III	ST239	1	-	t037	BEC	-	-	-	-	-	-	-	-	+	+	+	+	+	-	+	+	+	22
	C1	R4	Sa43	**R**	III	ST239	2	-	t037	BEC	-	-	-	-	-	-	-	-	+	+	+	+	+	-	+	-	-	19
	C1	R4	Sa67	**R**	III	ST239	4	A	t037	BEC	-	-	-	-	-	-	-	-	+	+	+	+	+	-	-	+	+	18
	C1	R4	Sa19	**R**	III	ST239	5	A	t037	BEC	-	-	-	-	-	-	-	-	+	-	+	+	+	-	-	+	-	7
	C1	R4	Sa31	**R**	III	ST239	6	A	t037	BEC	-	-	-	-	-	-	-	-	+	+	+	+	+	-	+	+	+	22
	C1	R4	Sa47	**R**	III	ST239	7	A	t037	BEC	-	-	-	-	-	-	-	-	+	+	+	+	+	-	+	-	+	20
	C1	R4	Sa48	**R**	III	ST239	7	A	t037	BEC	-	-	-	-	-	-	-	-	+	+	+	+	+	-	+	-	-	19
	C1	R4	Sa20	**R**	III	ST239	8	A	t037	BEC	-	-	-	-	-	-	-	-	-	-	+	+	+	-	-	+	-	1
	C1	R4	Sa57	**R**	III	ST239	9	A	t037	BEC	-	-	-	-	-	-	-	-	+	+	+	+	-	-	-	+	-	14
	C1	R4	Sa55	**R**	III	ST239	10	-	t037	BEC	-	-	-	-	-	-	-	-	+	+	+	+	-	-	-	+	+	15
	C1	R4	Sa86	**R**	III	ST239	12	B	t037	BEC	-	-	-	+	-	-	-	-	+	-	+	+	+	+	+	-	+	33
	C1	R4	Sa63	**R**	III	ST239	13	B	t037	BEC	-	-	-	-	-	-	-	-	+	+	+	+	+	-	+	-	+	20
	C1	R4	Sa66	**R**	III	ST239	14	B	t037	BEC	-	-	-	-	-	-	-	-	+	+	+	+	+	-	+	-	-	19
	C1	R4	Sa07	**R**	III	ST239	15	B	t037	BEC	-	-	-	-	-	-	-	-	+	+	+	+	+	-	-	+	+	18
	C1	R4	Sa18	**R**	III	ST239	15	B	t037	BEC	-	-	-	-	-	-	-	-	+	+	+	+	+	-	-	+	-	17
	C1	R4	Sa53	**R**	III	ST239	16	B	t037	BEC	-	-	-	-	-	-	-	-	+	+	+	+	+	-	-	-	-	16
	C1	R4	Sa36	**R**	III	ST239	17	-	t037	BEC	-	-	-	-	-	-	-	-	+	+	+	+	+	-	+	+	+	22
	C1	R4	Sa37	S	-	ST239	57	-	t037	-	-	-	-	-	-	-	-	-	+	+	+	+	+	-	+	-	+	20
	C1	R4	Sa52	S	-	ST239	58	-	t037	-	-	-	-	-	-	-	-	-	+	+	+	+	-	-	-	+	+	15
	C1	R4	Sa38	S	-	ST15	54	G	NT^i^	-	-	-	-	-	-	-	-	-	+	-	+	+	+	-	+	+	+	9
	C1	R4	Sa17	S	-	ST333	50	G	NT^i^	-	+	-	-	-	-	-	-	-	+	+	+	+	+	-	-	+	-	45
	C1	R4	Sa60	S	-	ST333	50	G	t084	-	-	-	-	-	-	-	-	-	+	+	+	-	+	-	+	-	-	12
	C1	R4	Sa06	S	-	ST333	53	G	t084	-	-	-	-	-	-	-	-	-	+	+	+	+	+	-	-	+	+	18
5	C1	R6	Sa46	S	-	ST101	31	-	t056	-	-	-	-	-	-	-	-	-	+	+	+	+	+	-	+	-	-	19
6	C1	R7	Sa26	S	-	ST1	35	E	t127	USA400^j^	-	-	-	+	-	-	-	-	+	-	+	+	+	+	-	+	-	32
	C1	R7	Sa27	S	-	ST1	35	E	t127	USA400^j^	-	-	-	+	-	-	-	-	+	-	+	+	+	-	-	+	+	31
	C1	R7	Sa16	S	-	ST1	36	E	t127	USA400^j^	-	-	-	+	+	-	-	-	+	+	+	+	+	+	-	+	-	39
	C1	R7	Sa25	S	-	ST1	30	-	t2279	-	-	-	-	+	-	-	-	-	+	+	+	+	+	+	+	+	+	38
	C1	R7	Sa10	S	-	ST1	48	-	t2279	-	-	-	-	+	-	-	-	-	+	+	+	+	+	+	-	+	+	37
7	C1	R12	Sa83	S	-	ST333	52	G	t084	-	-	-	-	+	-	-	-	-	+	+	+	+	+	+	+	+	+	38
8	C1	R14	Sa85	S	-	ST2383^g^	29	-	t2279	-	-	-	-	+	-	-	-	-	+	+	+	+	+	+	+	+	+	38
9	C2	R1	Sa81	**R**	II	ST105	25	-	t002	USA100	-	-	-	-	-	-	-	-	+	+	+	+	+	+	+	+	-	25
	C2	R1	Sa01	**R**	IV	ST2381^g^	18	C	t002	USA800	-	-	-	-	-	-	-	-	+	-	-	-	+	+	+	+	+	4
	C2	R1	Sa41	**R**	IV	ST5	20	C	NT^i^	USA800	-	-	-	-	-	-	-	-	+	+	+	+	+	-	+	-	-	19
	C2	R1	Sa58	**R**	IV	ST5	20	C	NT^i^	USA800	-	-	-	-	-	-	-	-	+	+	+	+	+	-	-	+	-	17
	C2	R1	Sa59	**R**	IV	ST5	21	C	NT^i^	USA800	-	-	-	-	-	-	-	-	+	+	+	+	+	-	+	+	+	22
	C2	R1	Sa82	**R**	IV	ST5	19	C	t002	USA800	-	-	+	-	-	-	-	-	+	+	+	+	+	+	+	+	+	41
	C2	R1	Sa54	**R**	IV	ST5	20	C	t002	USA800	-	-	-	-	-	-	-	-	+	+	+	+	+	-	-	+	-	17
	C2	R1	Sa79	**R**	IV	ST5	22	C	t002	USA800	-	-	-	-	-	-	-	-	+	+	+	+	+	-	-	+	+	18
	C2	R1	Sa30	**R**	IV	ST5	23	D	t002	USA800	-	-	-	-	-	-	-	-	+	+	+	+	+	-	-	+	-	17
	C2	R1	Sa33	**R**	IV	ST5	24	D	t002	USA800	-	-	-	-	-	-	-	-	+	+	+	+	+	-	+	+	+	22
	C2	R1	Sa69	**R**	IV	ST5	20	C	t267	USA800	-	-	-	-	-	-	-	-	+	+	+	+	+	-	+	-	+	20
	C2	R1	Sa76	**R**	IV	ST5	18	C	t6787	USA800	-	-	-	-	-	-	-	-	+	+	+	+	-	-	-	+	+	15
	C2	R1	Sa12	S	-	ST1635	40	F	t002	USA800^j^	-	-	-	-	-	-	-	-	+	+	+	+	+	-	-	+	+	18
	C2	R1	Sa51	S	-	ST1635	40	F	t002	USA800^j^	-	-	-	-	-	-	-	-	+	+	+	+	-	-	-	+	+	15
	C2	R1	Sa29	S	-	ST1635	41	F	t002	USA800^j^	-	-	-	-	-	-	-	-	+	+	+	+	+	-	-	+	-	17
	C2	R1	Sa87	S	-	ST5	44	F	t002	USA800^j^	-	-	+	+	-	+	-	-	+	+	+	+	+	+	+	+	+	44
	C2	R1	Sa80	S	-	ST5	27	-	t002	-	-	-	+	-	-	-	-	-	+	+	+	+	-	-	-	+	+	40
	C2	R1	Sa21	S	-	ST5	39	-	t002	-	-	-	-	-	-	-	-	-	+	+	+	+	+	-	+	+	+	22
	C2	R1	Sa05	S	-	ST5	45	-	t002	-	-	-	-	-	-	-	-	-	+	+	+	+	+	+	+	+	+	26
	C2	R1	Sa40	S	-	ST5	47	-	t002	-	-	-	-	-	-	-	-	-	+	+	+	+	+	-	+	+	+	22
	C2	R1	Sa13	S	-	ST5	42	F	t10548^h^	USA800^j^	-	-	-	-	+	-	-	-	+	+	+	+	+	-	-	+	+	29
	C2	R1	Sa23	S	-	ST5	40	F	t1277	USA800^j^	-	-	-	-	-	-	-	-	+	+	+	+	+	-	-	+	-	17
	C2	R1	Sa71	S	-	ST5	43	F	t214	USA800^j^	-	-	-	-	-	-	-	-	+	+	+	+	+	-	+	+	+	22
	C2	R1	Sa03	S	-	ST5	38	-	t2164	USA100^j^	-	-	-	-	-	+	-	-	+	-	-	-	+	-	-	+	+	27
	C2	R1	Sa56	S	-	ST5	46	-	t306	-	-	-	-	-	-	-	-	-	+	+	+	+	+	-	-	+	-	17
	C2	R1	Sa11	S	-	ST5	49	-	t9734	-	-	-	-	-	-	-	-	-	+	+	+	+	+	-	-	+	+	18
10	C2	R11	Sa61	S	-	ST2382^g^	37	-	t189	-	-	-	-	-	-	-	-	-	+	-	-	+	+	-	+	-	+	5
	C2	R11	Sa64	S	-	ST2382^g^	37	-	t189	-	-	-	-	-	-	-	-	-	+	+	+	+	+	-	-	-	-	16
11	C2	R2	Sa89	S	-	ST285	66	H	t021	USA1100^j^	-	-	-	-	-	-	-	-	+	+	+	+	+	+	+	+	+	26
	C2	R2	Sa15	S	-	ST30	62	H	t021	USA1100^j^	-	-	-	-	-	-	-	-	+	+	+	+	+	-	-	+	+	18
	C2	R2	Sa62	S	-	ST30	63	H	t318	USA1100^j^	-	-	-	-	-	-	-	-	+	+	+	+	+	+	-	-	+	23
	C2	R2	Sa34	S	-	ST30	64	H	t318	USA1100^j^	-	-	-	-	-	-	-	-	+	+	+	+	+	-	+	+	+	22
	C2	R2	Sa02	S	-	ST30	67	H	t318	USA1100^j^	-	-	-	-	-	-	-	-	+	+	+	+	+	-	+	+	+	22
	C2	R2	Sa70	S	-	ST30	67	H	t318	USA1100^j^	-	-	-	-	-	-	-	-	+	+	+	+	+	-	-	+	-	17
12	C2	R3	Sa04	S	-	NT^i^	NT^i^	NT^i^	t10550^h^	-	-	-	-	-	-	-	-	-	+	-	-	-	+	+	+	+	+	4
	C2	R3	Sa50	S	-	ST30	64	H	t1001	USA1100^j^	-	-	-	+	-	-	-	-	+	+	+	+	+	-	+	-	-	35
	C2	R3	Sa35	S	-	ST30	65	H	t433	USA1100^j^	-	-	-	+	-	-	-	-	+	+	+	+	+	-	+	-	+	36
	C2	R3	Sa42	S	-	ST30	65	H	t433	USA1100^j^	-	-	-	+	-	-	-	-	+	+	+	+	+	-	+	-	-	35
13	C2	R5	Sa08	S	-	ST45	59	-	t10550^h^	-	-	-	+	-	-	+	-	-	+	+	+	+	+	-	-	+	+	43
14	C2	R6	Sa09	S	-	NT^i^	NT^i^	NT^i^	t037	-	-	-	-	-	-	-	-	-	+	+	+	-	+	-	+	+	+	13
15	C2	R8	Sa24	S	-	ST6	28	-	t701	-	-	-	-	+	-	-	-	-	+	+	+	+	+	-	-	+	-	34
16	C2	R9	Sa28	S	-	ST5	42	F	t10548^h^	USA800^j^	-	-	-	-	-	-	-	-	+	+	-	+	-	-	-	-	-	10
17	C2	R10	Sa32	S	-	ST45	61	-	t1646	USA600^j^	-	-	+	-	-	+	-	-	+	+	+	+	+	-	-	-	+	42
18	C2	R13	Sa84	S	-	ST45	60	-	t065	-	-	-	-	-	-	+	-	-	+	+	+	+	+	+	+	+	+	28
19	C3	R4	Sa45	S	-	ST398	NT^i^	NT^i^	t1451	-	-	-	-	-	-	-	-	-	+	+	+	+	+	-	+	-	-	19
20	C4	R2	Sa49	S	-	ST97	33	E	t267	USA400^j^	-	-	-	-	-	-	-	-	+	+	+	+	+	-	+	+	-	21
	C4	R2	Sa72	S	-	ST669	34	E	t359	USA400^j^	-	-	-	-	-	-	-	-	+	-	+	-	+	+	+	-	-	6
	C4	R2	Sa78	S	-	ST97	56	-	t267	-	-	-	-	-	-	-	-	-	+	+	+	-	-	-	+	-	+	11
21	C4	R3	Sa14	S	-	ST120	70	-	t645	-	-	-	-	-	+	-	-	-	+	+	+	+	+	-	-	+	+	29
22	C4	R9	Sa44	S	-	ST97	32	E	t521	USA400^j^	-	-	-	-	-	-	-	-	+	+	+	+	+	-	+	-	+	20
Total:											1	0	6	13	4	5	0	0	88	74	81	78	80	19	49	62	58	

^a^OXA/CFO = resistant (R) or sensitive (S) to oxacillin/cefoxitin;

^b^E/P Clone = Epidemic/Pandemic Clone;

^c^ET = Exfoliative toxins;

^d^TSST = Toxic shock syndrome toxin;

^e^se = Staphylococcal enterotoxins;

^f^ *egc*= Enterotoxin gene cluster ( *seg, sei, sem, sen* and *seo*);

^g^ST2381, ^g^ST2382 and ^g^ST2383= new sequence types described;

^h^t10548 and ^h^t10550= new *spa* types describe;

^i^NT= non-typeable;

^j^USA100, ^j^USA400, ^j^USA600, ^j^USA800, ^j^USA1100 = MRSA clone related / PFGE analysis.

Twenty-six isolates (26/89 - 29%) exhibited the genotype C/R-9. Of these isolates, 21 (21/26 - 80,8%) exhibit ST (multilocus sequence type profile) 5, in which 10 MRSA isolates are related to the Pediatric clone (PC, also called USA800), harboring the following molecular characteristics: ST5, *spa* types t002 (5 isolates), t267, t6787, *spa* non-typeable (3 isolates), SCC *mec*IV, and grouped into PFGE clusters C or D. Four MSSA isolates within ST5 (t002/t10548/t1277/t214) and 3 MSSA isolates ST1635/t002 are also related to PC and were grouped together into PFGE cluster F and within genotype 9. One additional MRSA isolate (Sa01) was related to USA800/PC, even though with another ST (the newly described ST2381), grouped into PFGE cluster C, and also was classified as genotype 9.

One MRSA isolate (Sa81, ST105/t002-SCC *mec*II) and 1 MSSA isolate (Sa03, ST5/t2164) were individually related to the New York/Japan clone, or USA100 (ST5-SCC *mec*II) in a PFGE dendrogram previously obtained ( Andrade-Figueiredo and Leal-Balbino, 2016). These STs within genotype C/R-9 (ST105, ST1635, and ST2381) belong to the same clonal complex of ST5 isolates (CC5) since they have six matching loci.

Genotype C/R-16 consists of only one isolate, a MSSA with ST5 and in PFGE cluster F, and therefore, also related to PC/USA800 ( Table 3). Genotypes C/R-9 and C/R-16 differ only in their ITS-PCR profiling. While R1 (ITS-PCR profiling for genotype C/R-9) has 5 bands, R9 (ITS-PCR profiling for genotype C/R-16) has 4, and 3 of these bands are exactly at the same height. We can therefore suggest that both of these genotypes are related.

The genotype C/R-4 was observed in 23 isolates (23/89 - 26%), of which 17 (17/23 - 74%) are MRSA ST239/t037-SCC *mec*III, related to the Brazilian epidemic clone (BEC); 13 of these isolates were grouped into PFGE clusters A (7 isolates) or B (6 isolates). Two MSSA isolates with the genotype C/R-4 were also ST239/t037. In addition, 3 MSSA isolates that exhibit ST333 (t084 [2 isolates], *spa* non-typeable) and 1 MSSA isolate, ST15 and *spa* non-typeable, were genotyped C/R-4 and grouped together into PFGE cluster G ( Table 3).

The genotype C/R-11 was observed in 6 MSSA isolates (6/89 - 7%), in which 5 ST30 (t318 [4 isolates] and t021 [1]), and 1 isolate ST285/t021 are related to the Oceania Southwest Pacific clone (OSPC, also called USA1100 - ST30-SCC *mec*IV) and were grouped in PFGE cluster H. Similarly, the genotype C/R-12 was observed in 4 MSSA isolates (4/89 - 4%) MSSA isolates, of which 3 are ST30 (t433 [2 isolates] and t1001 [1]) and 1 (Sa4) is ST and PFGE non-typeable. These isolates are also related to the OSPC clone and were grouped into cluster H ( Table 3). ST30 and ST285 belong to the same clonal complex (CC30), as they have four or more similar MLST loci.

The genotype C/R-3 was observed in 6 isolates (6/89 - 7%), harboring STs ST239, ST25, ST30, ST333, and ST71. Two of these isolates were related to international clones: Sa73, classified as ST239/t037-SCC *mec*III is related to the BEC clone (PFGE cluster B); and Sa65 (ST30) is related to OSPC (cluster H).

All 5 MSSA isolates (5/89 - 6%) within ST1 exhibited the genotype C/R-6, of which 3 (ST1/t127) are related to the USA400 clone (ST1-SCC *mec*IV) and grouped into PFGE cluster E.

Three isolates (3/89 - 3%) were genotype C/R-20, of which two (Sa72 [ST669/t359] and Sa49 [ST97/t267]) were also related to the USA400 clone and grouped into cluster E. The genotype C/R-10 was observed in two (2/89 - 2%) MSSA isolates that exhibit a new ST (ST2382) and spa type t189.

## Discussion

*Staphylococcus aureus* is responsible for a broad spectrum of diseases in humans due to its ability to express several virulence factors, including enterotoxins, toxic shock syndrome toxin, and exfoliative toxins. Among them, SEs are the major cause of staphylococcal food poisoning ( Argudin et al., 2010). Ferry et al. (2005) described that *S. aureus* strains that cause sepsis, with or without shock, harbor at least one superantigen-encoding gene. Several diseases, including infectious endocarditis and food poisoning, have already been linked to *egc* ( Johler et al., 2015; Stach et al., 2016).

Different rates of toxigenic genes have been found in *S. aureus* clinical isolates from multiple countries, according to a number of investigations ( Song et al., 2016; De Carvalho et al., 2019). In our study, an elevated frequency of *S. aureus* clinical isolates comprising toxigenic genes was observed, especially those with a complete *egc*. All isolates related to the USA800/PC clone contained a complete *egc*, as well as other toxins, except for two isolates (Sa1 and Sa76) which exhibited only part of this cluster. According to Monecke et al. (2011), CC5 isolates carry the enterotoxin gene cluster, although partial deletions have been observed. 

Only a few studies have examined the frequency of toxigenic genes in *S. aureus* in Brazil, particularly in the North and Northeast regions and especially in clinical specimens ( Vasconcelos et al., 2011). In one study, 14% of MRSA strains from a university general hospital in Recife during 2002-2003 were related to the USA800 clone and harbored *egc*. Additionally, approximately 70% of MRSA strains were related to BEC, and none of them had toxigenic genes ( De Miranda et al., 2007).

Only the classic staphylococcal enterotoxins ( *sea-see*) were statistically associated with the resistance status of the isolates, and MSSA had the highest frequency of these superantigens. According to earlier research, MRSA makes up no more than 10% of colonizing strains, which suggests that MSSA is very common in colonizing and/or community-acquired infections ( Mehraj et al., 2016). The predominance of these genes in MSSA isolates raises concerns for community-associated infections because the classical enterotoxins are strongly associated with food poisoning ( *seb*), lethal sepsis ( *sec*), and infective endocarditis ( *sec*) ( Salgado-Pabón et al., 2013; Ahmad-Mansour et al., 2021).

The *eta* gene was found in only one isolate, and the low rates of isolates carrying the *eta* and *etb* genes responsible for SSS are in accordance with other studies, which have also demonstrated the low frequency of these genes in *S. aureus* isolates ( Becker et al., 1998, 2003). 

The *tst* gene, responsible for TSS, was observed in six isolates. Two of which were related to USA800 (MRSA Sa82 and MSSA Sa87), agreeing with the results of Durand et al. (2006) and Takano et al. (2008), and one isolate was related to USA600 (Berlin clone, BC, ST45-SCC *mec*IV), in accordance with the observations of Tenover et al. (2008) and King et al. (2016). Monecke et al. (2011) described that a similar CC45-MRSA-SCC *mec*IV strain, isolated in Australia, carries *sec*, *sel*, *tst*, and arginine catabolic mobile elements (ACME). Additionally, Portugal, Australia (WA MRSA-4), and Germany have all reported occasional cases of ST45-MRSA-SCCmecV. Most isolates tested in the study harbor *tst*, *sek,* and *seq* ( Aires-de-Sousa et al., 2008; Monecke *et al*., 2011).

The isolates from our study were from different clinical specimens. It is interesting to note that one isolate (MSSA Sa08, *tst* positive) was obtained from vaginal secretion. TSS was initially associated with the use of superabsorbent tampons in women with *S. aureus tst* producers on vaginal secretion; however, later, cases of non-menstrual TSS in the community and hospitals became prevalent ( Fitzgerald et al., 2001; Durand et al., 2006).

In the current study, we found significant genetic diversity among *S. aureus* isolates as well as a high frequency of toxigenic genes. Molecular typing techniques can be used to understand this diversity and how these strains are related. In some circumstances, where speed is required to identify a local outbreak and design containment plans, PCR-based genotyping approaches, such as coagulase gene typing and ITS-PCR, are fast and offer significant discriminatory power. According to Hunter and Gaston (1988), a discriminatory index greater than 0.90 can be interpreted as reliable and is thus desirable. However, even though PFGE has shown greater discriminatory power ( *DI* 0.99), the combination of PCR-based typing methods proved to be a useful and inexpensive procedure for conducting epidemiological surveys of *S. aureus* on a local or regional scale, even with a *DI* of 0.84. 

The analysis of PCR- *coa* identified five different amplicons. The 3´end coding region of the coa gene contains a series of repeating 81bp DNA sequences that differ in the number of tandem repeats. Since this region exhibits polymorphism, it is useful as a typing method ( Aarestrup et al., 1995).

We chose ITS-PCR as one of our genotyping methods in this study because of its practicality, cost-effectiveness, and alignment with the specific research objectives ( Liu et al., 2008). This technique has several advantages, such as simplicity, speed, and the ability to discern between closely related strains based on variations within the ITS region ( Boom et al., 1990; Gray et al., 2014). However, it is important to recognize that there are other genotyping techniques, such as ribotyping, that provide extensive information about the ribosomal RNA sequence and rely on the sequencing or hybridization of ribosomal RNA segments ( Bouchet et al., 2008).

On the other hand, ITS-PCR focuses specifically on the amplification and analysis of a specific genomic region, the ITS region of the ribosomal RNA operon. This targeted approach allows efficient screening of large collections of strains and can provide valuable information about the genetic diversity and lineage of strains based on variations within the ITS region ( Ryberg et al., 2011; Lian and Zhao, 2016). By employing ITS-PCR in conjunction with other molecular typing techniques such as coagulase typing, PFGE, *spa* typing and SCC *mec* genotyping, we were able to obtain comprehensive information on the molecular epidemiology and clonal relationships between the *S. aureus* strains under investigation.

In this study, C/R-9 was the most frequent genotype observed among isolates. All 26 isolates in C/R-9 were associated with Clonal Complex 5, grouping all USA800/PC and USA100 clones. Thus, the results indicate that the C/R combination was able to distinguish USA800/PC and USA100 clones from other strains. Only one isolate related to the USA800 clone (Sa28 ST5/ newly described t10548, cluster F) exhibited a different genotype, C/R-16, but this genotype appears to be related to C/R-9.

The BEC represents a multidrug-resistant lineage described in Brazil in 1992, linked to hospital-acquired infections (HA-MRSA), which was widely distributed in Brazil and later in other countries. However, in the first ten years of the 2000s, there was an increase in “imported” clones, such as the Pediatric clone (PC), the New York/Japan Clone, and other less common lineages ( Andrade et al., 2020). 

Since then, BEC prevalence appears to be decreasing, while infections by community-acquired (CA-MRSA) clones, such as PC and OSPC, have been steadily increasing. Complete BEC substitution has already been reported in some hospitals ( Chamon et al., 2017; Monteiro et al., 2019), and CA-MRSA clones appear to be becoming more common across Brazil ( Romero and Cunha, 2021). Historically, there have been distinctions between the epidemiologies of HA-MRSA and CA-MRSA, with CA-MRSA typically being linked to more virulent infections and infecting patients who have few or no risk factors. However, these distinctions are becoming less clear today ( Lee et al., 2018).

The genotype C/R-4 grouped 17 of 19 (89,5%) BEC and related clones, including all five BEC MRSA isolates from ICU (hospital 1), as well as distinguishing these from other epidemic/pandemic clones such as USA800/PC, USA100, USA1100, USA400, and USA 600. Only two BEC clones exhibited a diverse C/R pattern. The genotype C/R-4 also grouped 3 isolates within ST333 ( *spa* nontypeable, t084 [2 isolates], cluster G) and 1 isolate ST15 ( *spa* nontypeable, cluster G), both CC15.

All isolates within ST1 (Cluster E, CC1) were grouped into the genotype C/R-6 association. Coagulotype and ITS-PCR analyses were capable of distinguishing these isolates from others also clustered into PFGE cluster E and related to USA400 that exhibited ST669 and ST97, both STs from CC97. The genotypes C/R-11 and C/R-12 were able to group all isolates related to clone USA1100, except for isolate Sa65 (C/R-3).

We observed a more robust correlation between *coa*-PCR/ITS-PCR and PFGE/MLST patterns in MRSA isolates. The C/R association allowed us to observe the clonal spread of MRSA and MSSA within the main hospital analyzed (hospital 1). Patients from these isolates were dispersed throughout various hospital wings. Additionally, we discovered closely related isolates between hospital 1’s isolates and all four of hospital 2’s isolates.

No one genotyping technique that is now available is thought to be best for epidemiological studies. Every circumstance is unique, so it is important to assess the benefits and drawbacks of each technique both separately and collectively in order to choose the best methodology based on the targets and objectives outlined in each study.

Through combining coagulotype and ITS-PCR analysis, which showed a relationship with PFGE genotype and MLST as well as a minor correlation with *spa* typing, we found a high genetic diversity among the isolates in our study and observed clonal spread of MRSA and MSSA in hospital settings. It is important to emphasize that this specific association between techniques may be practical, quick, and affordable for initial epidemiological investigations in hospitals or local outbreaks, thus becoming an interesting strategy for countries and institutions with fewer resources.

We emphasize the need for further studies for epidemiological surveillance of MRSA and MSSA due to the change in *S. aureus* epidemiology and the growing threat of this pathogen to hospital and community environments.

## Supplementary material

The following online material is available for this article:

Table S1 - Clinical specimens and oxacillin/cefoxitin resistance of *Staphylococcus aureus* obtained from hospitals in Recife, Brazil.

Table S2 - Toxin gene profiling and data collection. Sheet 1 = toxin profile arrangement and counting; Sheet 2 = toxin profiles and toxin gene PCR findings for all isolates; Sheet 3 = MRSA isolates results; Sheet 4 = MSSA isolates results.
