# Supplementary material for: Comparison of genotyping methods and toxin gene profiles of Staphylococcus aureus isolates from clinical specimens
Source: Genet Mol Biol. 2023 Dec 22;46(4):e20220321. doi: 10.1590/1678-4685-GMB-2022-0321 (PMC10789239; doi:10.1590/1678-4685-GMB-2022-0321)
Supplement: Table S1 - [file 1415-4757-GMB-46-04-e20220321-s1.pdf]

*Genetics and Molecular Biology*
https://doi.org/10.1590/1678-4685-GMB-2022-0321

# Supplementary Material to "Comparison of genotyping methods and toxin gene profiles of *Staphylococcus aureus* isolates from clinical specimens"

**Table S1 -** Clinical specimens and oxacillin/cefoxitin resistance of *Staphylococcus aureus* obtained from hospitals in Recife, Brazil.

| Isolate | Clinical specimen | Hospital* | Hospital Unit | Oxa/Cefo[a] |
|---|---|---|---|---|
| Sa01 | Urine | 1 | OC[b] | **R** |
| Sa02 | Urethral secretion | 1 | OC | S |
| Sa03 | Vaginal secretion | 1 | OC | S |
| Sa04 | Urine | 1 | OC | S |
| Sa05 | Urine | 1 | OC | S |
| Sa06 | Urine | 1 | OC | S |
| Sa07 | Urine | 1 | OC | **R** |
| Sa08 | Vaginal secretion | 1 | OC | S |
| Sa09 | Sperm | 1 | OC | S |
| Sa10 | Urethral secretion | 1 | OC | S |
| Sa11 | Tip Catheter | 1 | Ward | S |
| Sa12 | Urine | 1 | OC | S |
| Sa13 | Urine | 1 | OC | S |
| Sa14 | Urine | 1 | OC | S |
| Sa15 | Urine | 1 | Ward | S |
| Sa16 | Urine | 1 | OC | S |
| Sa17 | Urine | 1 | OC | S |
| Sa18 | Urine | 1 | Ward | **R** |
| Sa19 | Urine | 1 | OC | **R** |
| Sa20 | Blood | 1 | ICU | **R** |
| Sa21 | Urine | 1 | Ward | S |
| Sa22 | Urine | 1 | OC | **R** |
| Sa23 | Urine | 1 | OC | S |
| Sa24 | Prosthesis Secretion | 1 | Ward | S |
| Sa25 | Wound Secretion | 1 | OC | S |
| Sa26 | Tip Catheter | 1 | Ward | S |
| Sa27 | Wound Secretion | 1 | OC | S |
| Sa28 | Urine | 1 | OC | S |
| Sa29 | Urine | 1 | Ward | S |
| Sa30 | Tissue Fragment | 1 | Ward | **R** |
| Sa31 | Bone Fragment (Tibia) | 1 | Ward | **R** |
| Sa32 | Tip Catheter | 1 | Ward | S |
| Sa33 | Surgical Wound Secretion | 1 | Ward | **R** |
| Sa34 | Blood | 1 | OC | S |
| Sa35 | Ocular Secretion | 1 | Ward | S |
| Sa36 | Tracheal Secretion | 1 | ICU | **R** |
| Sa37 | Ulcer Secretion | 1 | Ward | S |
| Sa38 | Bone Fragment | 1 | Ward | S |
| Sa39 | Tip Catheter | 1 | Ward | **R** |
| Sa40 | Thigh Abscess | 1 | Ward | S |
| Sa41 | Surgical Wound Tissue | 1 | Ward | **R** |
| Sa42 | Cavity of Tibia Secretion | 1 | Ward | S |
| Sa43 | Tip Catheter | 1 | Ward | **R** |
| Sa44 | Surgical Wound | 1 | Ward | S |
| Sa45 | Thoracic Drain Secretion | 1 | Ward | S |

| Isolate | Clinical specimen | Hospital* | Hospital Unit | Oxa/Cefo[a] |
|---|---|---|---|---|
| Sa46 | Oropharynx | 1 | OC | S |
| Sa47 | Tip Catheter | 1 | ICU | **R** |
| Sa48 | Ulcer Secretion | 1 | OC | **R** |
| Sa49 | Oropharynx Secretion | 1 | OC | S |
| Sa50 | Nasal secretion | 1 | OC | S |
| Sa51 | Tip Catheter | 1 | Ward | S |
| Sa52 | Tip Catheter | 1 | Ward | S |
| Sa53 | Surgical Wound Tissue | 1 | Ward | **R** |
| Sa54 | Tip Catheter | 1 | Ward | **R** |
| Sa55 | Oropharynx Exudate | 1 | ICU | **R** |
| Sa56 | Tip Drain | 1 | Ward | S |
| Sa57 | Forearm Purulent Exudate | 1 | Ward | **R** |
| Sa58 | Hip Bone Fragment | 1 | Ward | **R** |
| Sa59 | Surgical Wound Secretion | 1 | Ward | **R** |
| Sa60 | Wound Secretion | 1 | Ward | S |
| Sa61 | Tip Drain | 1 | Ward | S |
| Sa62 | Knee Abscess Secretion | 1 | Ward | S |
| Sa63 | Tip Catheter | 1 | Ward | **R** |
| Sa64 | Abdominal Collection | 1 | Ward | S |
| Sa65 | Oropharynx | 1 | Ward | S |
| Sa66 | Tracheal Secretion | 1 | ICU | **R** |
| Sa67 | Surgical Wound Secretion | 1 | Ward | **R** |
| Sa68 | Ulcer Secretion | 1 | Ward | S |
| Sa69 | Tissue Fragment | 1 | Ward | **R** |
| Sa70 | Secretion of Axilla | 1 | OC | S |
| Sa71 | Fistula Secretion | 1 | OC | S |
| Sa72 | Surgical Wound Secretion | 1 | Ward | S |
| Sa73 | Tip Catheter | 1 | Ward | **R** |
| Sa74 | Catheter Secretion | 1 | Ward | S |
| Sa75 | Blood | 1 | Ward | S |
| Sa76 | Frontal Lesion Secretion | 1 | OC | **R** |
| Sa77 | Ocular Secretion | 1 | Ward | S |
| Sa78 | Oropharynx | 1 | OC | S |
| Sa79 | Tip Catheter | 1 | Ward | **R** |
| Sa80 | Surgical Wound Secretion | 1 | OC | S |
| Sa81 | Blood | 3 | Ward | **R** |
| Sa82 | Ocular Secretion | 2 | Ward | **R** |
| Sa83 | Surgical Wound | 3 | CA[c] | S |
| Sa84 | Tracheal Secretion | 3 | CA | S |
| Sa85 | Blood | 3 | CA | S |
| Sa86 | Secretion/ PID[d] | 2 | PIPD[e] | **R** |
| Sa87 | Tracheal Secretion | 2 | ICU | S |
| Sa88 | Blood | 3 | CA | S |
| Sa89 | Tissue Lesion | 2 | OC | S |

*Hospital= general university hospital (hospital 1)/ second general university hospital (hospital 2)/ cardiology hospital (hospital 3)

[a]Oxa/Cefo= Oxacillin/Cefoxitin Resistance;

[b]OC= Outpatient Clinic;

[c]CA= Cardiology Emergency.

[d]PID= Parasitic/Infectious Disease;

[e]PIPD= Pavilion of Infectious and Parasitic Diseases;

Isolates that are *mec*A positive were considered oxacillin-susceptible and *mec*A-positive *S. aureus* (OS-MRSA) (Andrade-Figueiredo; Leal-Balbino, 2016).
